# Aurora A kinase regulates non-homologous end-joining and poly(ADP-ribose) polymerase function in ovarian carcinoma cells

**DOI:** 10.18632/oncotarget.18970

**Published:** 2017-07-05

**Authors:** Thuy-Vy Do, Jeff Hirst, Stephen Hyter, Katherine F. Roby, Andrew K. Godwin

**Affiliations:** ^1^ Department of Pathology and Laboratory Medicine, University of Kansas Medical Center, Kansas City, KS, USA; ^2^ Anatomy and Cell Biology, University of Kansas Medical Center, Kansas City, KS, USA; ^3^ University of Kansas Cancer Center, Kansas City, KS, USA

**Keywords:** Aurora A kinase, ovarian cancer, PARP, alisertib, double-strand break

## Abstract

Ovarian cancer is usually diagnosed at late stages when cancer has spread beyond the ovary and patients ultimately succumb to the development of drug-resistant disease. There is an urgent and unmet need to develop therapeutic strategies that effectively treat ovarian cancer and this requires a better understanding of signaling pathways important for ovarian cancer progression. Aurora A kinase (AURKA) plays an important role in ovarian cancer progression by mediating mitosis and chromosomal instability. In the current study, we investigated the role of AURKA in regulating the DNA damage response and DNA repair in ovarian carcinoma cells. We discovered that AURKA modulated the expression and activity of PARP, a crucial mediator of DNA repair that is a target of therapeutic interest for the treatment of ovarian and other cancers. Further, specific inhibition of AURKA activity with the small molecule inhibitor, alisertib, stimulated the non-homologous end-joining (NHEJ) repair pathway by elevating DNA-PKcs activity, a catalytic subunit required for double-strand break (DSB) repair, as well as decreased the expression of PARP and BRCA1/2, which are required for high-fidelity homologous recombination-based DNA repair. Further, AURKA inhibition stimulates error-prone NHEJ repair of DNA double-strand breaks with incompatible ends. Consistent with *in vitro* findings, alisertib treatment increased phosphorylated DNA-PKcs(pDNA-PKcs^T2609^) and decreased PARP levels *in vivo*. Collectively, these results reveal new non-mitotic functions for AURKA in the regulation of DNA repair, which may inform of new therapeutic targets and strategies for treating ovarian cancer.

## INTRODUCTION

Ovarian cancer (OC) is the most lethal gynecologic malignancy in the Western world, with an estimated 22,280 new cases and 14,240 deaths in the U.S. for 2016 [1]. Although most patients initially respond to surgical debulking and chemotherapy, the majority will experience recurrent disease that has become drug resistant [1, 2]. Oncology drugs lead other therapeutic areas in clinical development [3], but only 7% of drugs that enter Phase I clinical trials successfully attain marketing approval [4]. These statistics suggest that there is room for improvement in our approach to steering oncology drugs from the bench to the bedside.

*AURKA* was first discovered in *Drosophila melanogast*er as a gene that regulates mitotic spindle function [5] and has since been the focus of numerous studies documenting its essential roles in mitotic entry, centrosome function and bipolar spindle assembly [6]. AURKA is thought to play an important role in ovarian tumor biology, because activation of this kinase either by genomic amplification or increased expression is a common feature of ovarian carcinoma cell lines and primary tumors [7–13]. Further, high levels of active AURKA in ovarian tumors are also associated with supernumerary centrosomes and overall decreased survival [14].

Two small molecule inhibitors specific for AURKA, alisertib (Takeda Pharmaceuticals) and MK-5108 (Merck Research Laboratories), have been evaluated in clinical trials for the treatment of OC. Preliminary Phase I results for MK-5108 revealed activity as a single agent, as well as, in combination with docetaxel for the treatment of advanced solid tumors [15]. Alisertib (MLN8237) exhibits exquisite specificity for AURKA with >200-fold greater selectivity for AURKA than AURKB [16]. Using an orthotopic mouse model, we reported that alisertib specifically inhibited AURKA activity *in vivo* and exerts ovarian tumor growth inhibition (TGI) as a single agent [17]. Further, alisertib and paclitaxel combination therapy TGI was even more potent than that observed for monotherapy [17]. Alisertib showed modest effects for platinum-resistant and -refractory OC [18] when used as a single agent, and is currently in Phase II clinical trials in combination with paclitaxel [19, 20]. Early results from a Phase I/II alisertib and paclitaxel trial for ovarian and breast cancer reveal partial response in eight patients and stable disease for three patients [19]. The fact that alisertib, as a single agent or as part of a combination therapy regimen showed clinical activity in a subset of patients, underscores the need to improve our understanding of AURKA-regulated pathways that mediate tumor progression, including novel non-mitotic functions [17, 21–26].

While the role of AURKA in regulating mitosis has been extensively studied, little is known about the function of this kinase in mediating DNA repair and the DNA damage response (DDR). AURKA regulation of genomic instability has been linked to interactions with the caretakers of global chromosomal stability, BRCA1 and BRCA2. In the context of BRCA2, Yang et al [27] reported a functional interaction between AURKA and BRCA2 in sporadic disease and showed that AURKA inhibition of BRCA2 expression *in vitro* perturbs the DDR promoting cell cycle progression and genomic instability [27]. Analyses of 223 high-grade serous carcinomas uncovered an inverse correlation between AURKA and BRCA2 protein expression, with high AURKA to BRCA2 expression ratios predicting poor survival [27].

An inverse relationship between AURKA/B and BRCA1/2 has also been reported in vitro where silencing of *AURKA/B* by shRNA resulted in elevated expression of *BRCA1/2* [28]. Further, downregulation of *AURKA/B* inhibited aberrant cytokinesis and diminished cell multinuclearity and chromosome tetraploidy, while a knockdown of *BRCA1/2* expression had the opposite effect. Consistent with these observations, shRNA-mediated silencing of *AURKA* inhibited growth, while silencing of *BRCA1/2*, increased growth of tumor xenografts in mice [28].

AURKA may mediate chromosomal instability in tumor cells by regulating error-prone NHEJ DNA repair. *In vitro* studies in breast cancer cells revealed that *AURKA* overexpression diminished recruitment of RAD51 to sites of DSBs, which disrupted repair of DNA damage through the high-fidelity homologous recombination (HR)-dependent mechanism, thereby favoring the NHEJ pathway [25]. Moreover, loss of RAD51 recruitment to sites of DSBs required PLK1 inhibition of CHK1 activity [25].

Error-prone NHEJ results in chromosomal translocations and rearrangements [29, 30], leading to genomic instability. NHEJ is initiated when Ku80-Ku70 binds to DNA ends and recruits DNA-PKcs. DNA ends are then processed by several proteins, including Artemis, the polynucleotide kinase, and members of the polymerase X family [31–35], before ends are finally joined by ligase IV, which is part of a complex containing XRCC4 and Cernunos/Xlf [36–38].

Poly(ADP-ribose) polymerase 1 (PARP1) is a nuclear enzyme, which plays a critical role in DNA repair, including NHEJ. PARP1 (hereafter referred to as PARP) binds to damaged DNA and, when activated, produces poly(ADP-ribose) [pADPr] chains that binds covalently to chromatin proteins and to PARP itself, altering protein function [39–43]. A number of PARP inhibitors (PARPis) [e.g., rucaparib, niraparib, veliparib and talozaparib] are currently in clinical trials for the treatment of OC, and promising results led the Food and Drug Administration to approve olaparib (Lynparza) and to designate rucaparib as a Breakthrough Therapy [44]. PARPis were designed to target *BRCA*-mutated tumors since HR-based DNA repair is disrupted in these tumors [44]. Approximately 50% of high-grade serous ovarian carcinomas exhibit changes in genes (not limited to *BRCA1/2*) predicted to result in HR deficiency and these tumors are described as possessing ‘BRCAness’ [13, 45–48]. Patel *et al* [49] proposed a model in which PARPi is cytotoxic to ovarian carcinoma cells, because PARP inhibition stimulates NHEJ, thereby resulting in lethal genomic instability. Notably, PARPi stimulated error-prone NHEJ by activating DNA-PKcs only in HR-deficient and not in HR-proficient cells [49].

The current study tests the hypothesis that AURKA regulates the DDR and DNA repair pathways in ovarian carcinoma cells. Inhibition of AURKA activity significantly diminished cell growth, activated DNA-PKcs and decreased PARP expression and activity. Consistent with these observations, alisertib treatment also stimulated error-prone NHEJ DNA repair. Moreover, AURKA inhibition also decreased the expression of BRCA1 or BRCA2 and increased pH2AX^S139^ levels, suggesting that impaired HR pathway function and induction of DNA double-strand breaks (DSBs), respectively. These studies also promoted evaluation of combining alisertib and rucaparib to potentially extend the number of ovarian cancer patients with HR competent tumors who might benefit from a PARP inhibitor. Collectively, our findings reveal new functions for AURKA in the regulation of DNA repair, which shed new light on the complexity of AURKA-regulated pathways involved in the DDR.

## RESULTS

Overexpression of *AURKA* has been shown to disrupt HR, thereby stimulating NHEJ[25], yet our knowledge of AURKA regulation of error-prone NHEJ repair remains incomplete. Given the success of PARPis for the treatment of OC, particularly HR-deficient cancers, we explored the potential connection of AURKA activation on cell growth, the DDR and DNA repair in PARPi-sensitive (HR-deficient) and PARPi-resistant (HR-proficient) ovarian carcinoma cells.

### AURKA inhibits PARPi-sensitive and -resistant ovarian carcinoma cell growth and clonal survival

To understand the effects of AURKA inhibition, in the context of HR-proficiency and HR-deficiency, we employed a large panel of established ovarian carcinoma cell lines, including PEO1, SKOV3ip2, PEO4, OVCA429, MDAH, A2780, OVCAR5 and OVCAR10. We first treated this panel of cell lines with the PARPi, rucaparib, and evaluated cell viability to determine the comparative sensitivity as an indication of HR status for each cell line. Results from this analysis showed a range of sensitivities among the eight ovarian cell lines tested (Supplementary Table 1), with PEO1, a *BRCA2*-mutated and HR-deficient cell line [50], being the most sensitive (IC_50_ = 0.324 μmol/L); PEO4 and SKOV3ip2 (which have no deleterious *BRCA1/2* mutations) [50, 51] were resistant to rucaparib; OVCA429 (which has no deleterious *BRCA1/2* mutations) [51] exhibited modest sensitivity to rucaparib (IC_50_ = 5.5 μmol/L). Primary normal human ovarian surface epithelial (HOSE) cells were also treated with rucaparib and exhibited resistance to PARPi (Supplementary Table 1).

**Figure 1:**
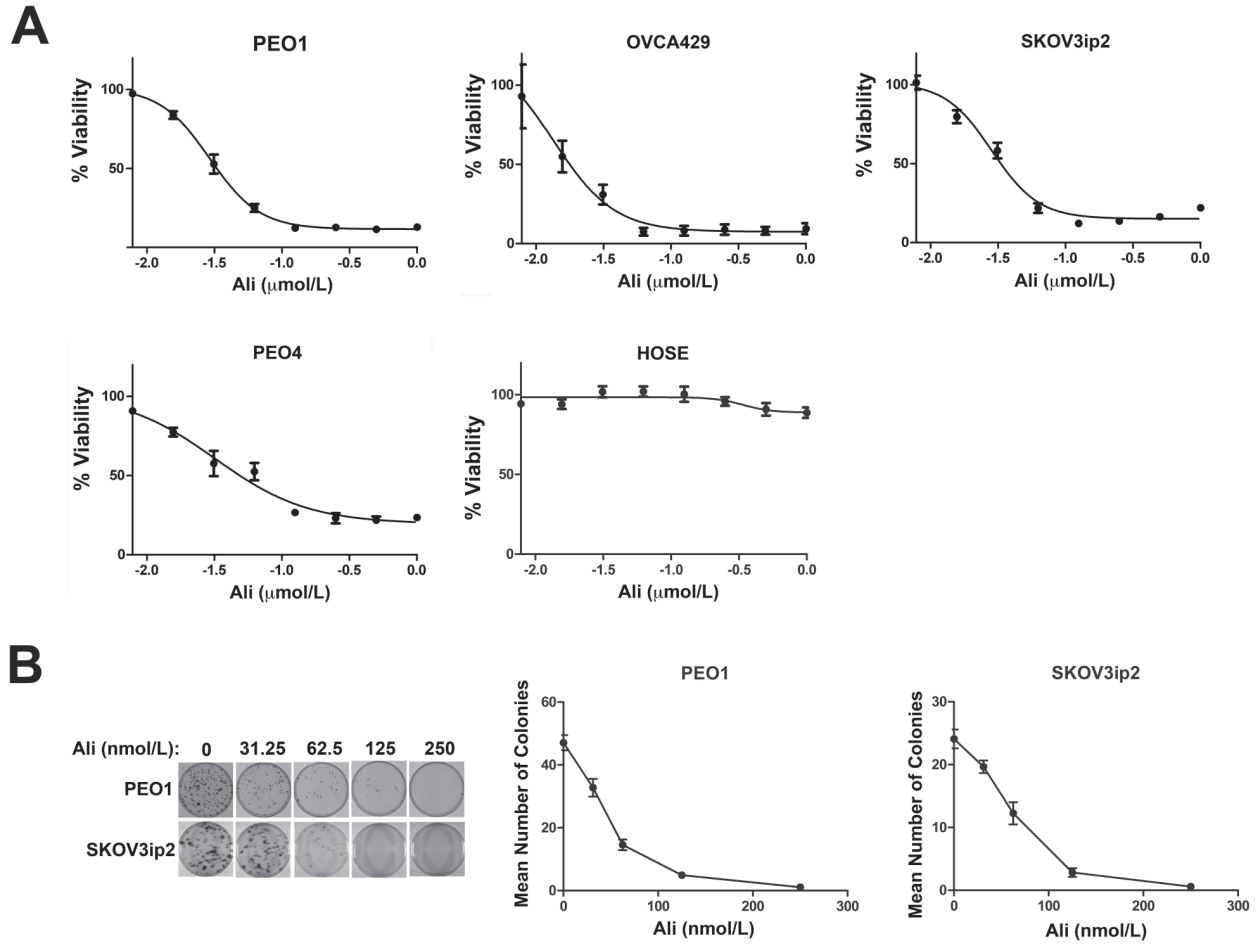
Inhibition of AURKA activity diminishes the growth and clonogenic survival of ovarian carcinoma cells **A**. PEO1, PEO4, OVCA429 and SKOV3ip2, in addition to normal HOSE cells, were treated for seven days with increasing concentrations of alisertib (0.00781, 0.0156, 0.0313, 0.0625, 0.125, 0.250, 0.50, and 1.0 μmol/L) and then viability was assayed using Cell Titer Blue. Graphs that depict the mean percentage of viable cells ± SEM for each concentration of alisertib from three independent experiments are shown. The effect of AURKA inhibition on clonogenic survival of ovarian carcinoma cells was also analyzed by treatment with increasing concentrations of alisertib (0.0313, 0.0625, 0.125, and 0.250 μmol/L). **B**. Cells were stained with crystal violet and graphs depicting the mean number of PEO1 and SKOV3ip2 colonies ± SEM for each alisertib concentration from three independent experiments are shown. Cells were treated with vehicle (DMSO) as a control.

We next examined the effect of alisertib treatment for seven days on ovarian carcinoma cell growth using PEO1, OVCA429, PEO4 and SKOV3ip2 cells (Figure 1A & Table 1), which exhibit high sensitivity, moderate sensitivity and resistance to rucaparib, respectively. We also treated HOSE cells with alisertib to determine the effects of this AURKA-specific small molecule inhibitor on normal cells. Alisertib potently inhibited the growth of PARPi-sensitive and PARPi-resistant ovarian carcinoma cells but had very little effect on the growth of normal HOSE cells (Figure 1A & Table 1). Similarly, treatment with nanomolar concentrations of alisertib dramatically decreased clonal survival in both PEO1 and SKOV3ip2 (Figure 1B) cells in a dose-dependent manner.

**Table 1:** Ovarian carcinoma cell line IC_50_ values for alisertib

Cell Line	Mean IC_50_ ± SE (nM)
PEO1	32.9 ± 2.69
OVCA429	17.7 ± 5.18
PEO4	47.8 ± 11.5
SKOV3ip2	31.1 ± 3.52
HOSE	NA

We previously reported alisertib had modest effects on ovarian carcinoma cell growth after a three-day treatment period [17], but our current findings suggest that, similar to PARPi, longer exposure to this small molecule inhibitor is required to see more dramatic effects on cell growth.

### AURKA inhibition alters the expression of DDR proteins

**Figure 2:**
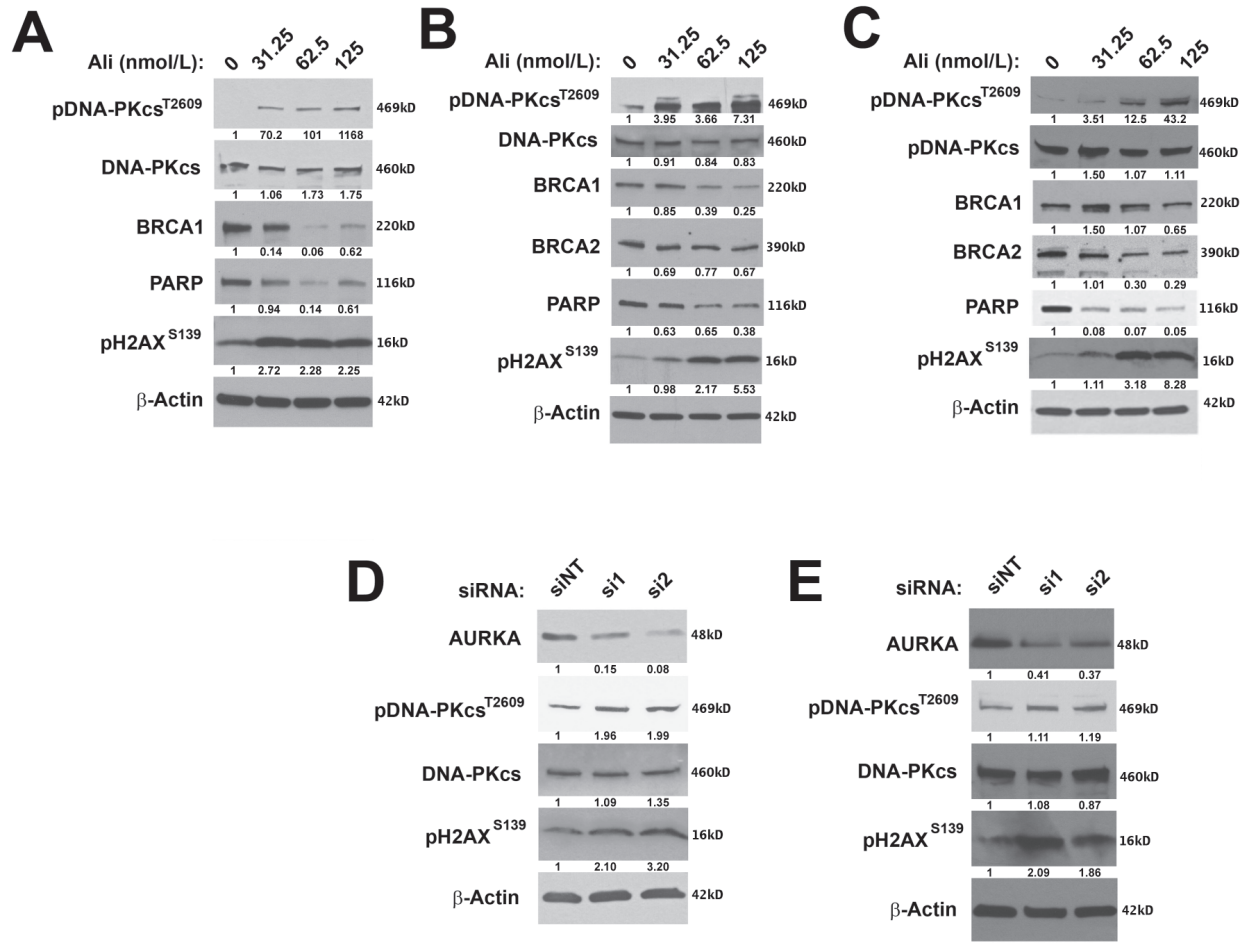
Inhibition of AURKA activity stimulates the NHEJ pathway and decreases the expression of HR proteins **A**. PARPi-sensitive PEO1 and **B**. PARPi-resistant PEO4 and **C**. SKOV3ip2 cells were treated with vehicle (DMSO) or alisertib (31.25, 62.5, or 125 nmol/L) for 48 h and then total protein lysates were immunoblotted with antibodies against pDNA-PKcs^T2609^, total DNA-PKcs, BRCA1, BRCA2, PARP1, pH2AX^S139^, and β-Actin (loading control). Representative immunoblots are shown. To evaluate the effects of RNAi-mediated silencing of *AURKA*, **D**. PEO1 and **E**. SKOV3ip2 cells were transiently transfected with non-targeting (siNT) or *AURKA*-specific siRNAs (si1 and si2) for 48 h, and then total protein lysates were immunoblotted with antibodies against AURKA, pDNA-PKcs^T2609^, total DNA-PKcs, pH2AX^S139^, and -actin (loading control). Representative immunoblots are shown with densitometry measurements normalized to both β-actin and experimental controls under each blot.

To determine the effects of inhibiting AURKA activity on the DDR in ovarian carcinoma cells, we first analyzed the effect of alisertib treatment on the expression of important HR and NHEJ pathway signaling molecules, in PARPi-sensitive (PEO1) and -resistant (PEO4 and SKOV3ip2) cell lines. Alisertib treatment at nanomolar concentrations that are not expected to yield off-target effects [52], decreased the expression of PARP in a dose-dependent manner in all cell lines assayed, PEO1, PEO4 and SKOV3ip2 (Figure 2A). Alisertib treatment also decreased the expression of the HR pathway proteins, BRCA1 or BRCA2, in PEO1 and PEO4 cells or SKOV3ip2 (Figure 2A) cells, respectively.

To evaluate the potential effects of AURKA inhibition on the NHEJ pathway, we analyzed the phosphorylation of DNA-PKcs at Thr2609, which is required for DSB repair by NHEJ [53]. Alisertib treatment increased pDNA-PKcs^T2609^ levels in PARPi-sensitive (PEO1) and -resistant (SKOV3ip2 and PEO4) ovarian carcinoma cells (Figure 2A). Pharmacological inhibition of AURKA activity also increased pH2AX^S139^ levels in all cell lines assayed (Figure 2A). Collectively, these observations suggest that sustained inhibition of AURKA activity induces DNA damage, attenuates the HR pathway, and stimulates the NHEJ pathway.

To further confirm the specificity of the effect of AURKA inhibition on the NHEJ pathway, we transfected PEO1 and SKOV3ip2 cells with two independent siRNAs targeting *AURKA* and a non-targeted (NT) control. Since AURKA is essential for ovarian carcinoma cell survival, highly efficient *AURKA* knockdown is lethal to cells. Therefore, conditions resulting in partial knockdown of AURKA expression were employed, showing that depletion of AURKA expression increased phosphorylation of DNA-PKcs and H2AX in PEO1 and SKOV3ip2 cells (Figure 2B).

Collectively, these findings indicate that AURKA inhibition stimulates the NHEJ pathway by increasing DNA-PKcs activity and decreasing the expression of HR proteins, BRCA1/2. AURKA inhibition also elevated pH2AX^S139^ levels suggesting the induction of DNA DSBs.

### Inhibition of AURKA activity increases pH2AXS139 foci and error-prone NHEJ DNA repair

**Figure 3:**
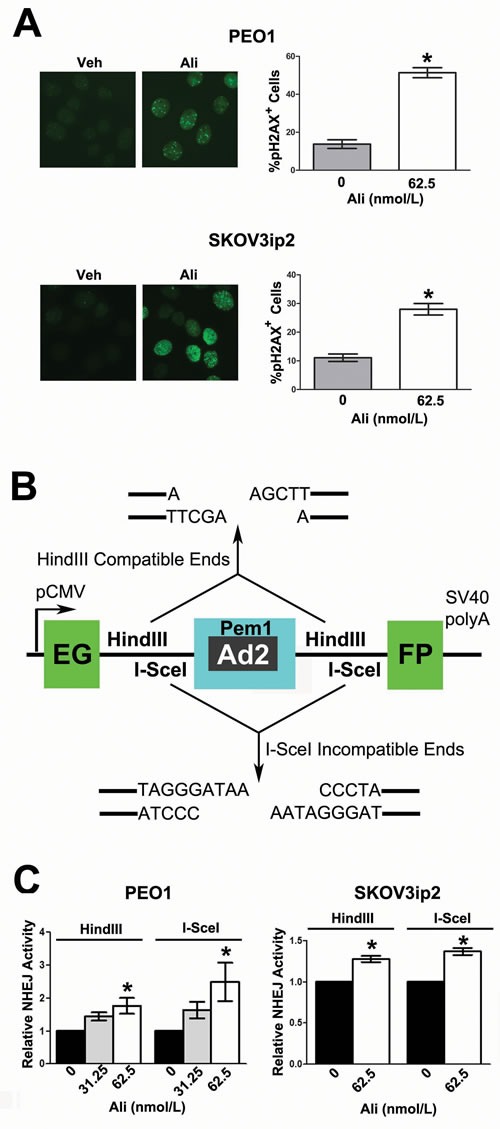
Inhibition of AURKA activity induces the formation of pH2AXS139 foci and stimulates error-prone NHEJ **A**. PEO1 and SKOV3ip2 cells were treated with 62.5 nmol/L alisertib for 24 h before pH2AX^S139^ nuclear foci were enumerated. Graphs that depict the mean percentage of cells positive for ≥ 10 pH2AX^S139^ foci ± SEM (relative to vehicle-treated control) from three independent experiments are shown. Bars labeled with asterisks are statistically significant as analyzed by Wilcoxon matched-pairs signed rank test (*P < 0.05). To determine the functional effects of AURKA inhibition on NHEJ DNA repair, ovarian carcinoma cells were subjected to a validated NHEJ assay after alisertib treatment. A schematic of the **B**. *pEGFP-Pem1-Ad2-GFP* harboring the *EGFP* open-reading frame, which is interrupted by a Pem1 intron containing an adenoviral exon (Ad2) that is flanked on either side by *HindIII* and *I-SceI* restriction sites. Digestion with *HindIII* generates compatible DNA ends, while *I-SceI* digestion generates incompatible DNA ends. Cleavage with either *HindIII* or *I-SceI* creates a DNA DSB and removes the Ad2 exon; successful DNA repair results in recircularization of the plasmid, generating a functional EGFP. **C**. PARPi-sensitive PEO1 and PARPi-resistant SKOV3ip2 cells were transiently transfected with *peEGFP-Pem1-Ad2* digested with either *HindIII* or *I-SceI*, in addition to an *RFP* plasmid (which served as a control for transfection efficiency). Cells were also treated with vehicle (DMSO) or alisertib (62.5 nmol/L) for 48 h, and then the percentage of EGFP^+^ and RFP^+^ cells was determined by flow cytometry. Graphs depicting the mean number of EGFP^+^ cells normalized to the number of RFP^+^ cells transfected with the *HindIII*- or *I-SceI*-digested DNA substrates ± SEM (relative to vehicle-treated control) from three independent experiments are shown. Bars labeled with asterisks are statistically significant as analyzed by one-way ANOVA followed by Tukey's multiple comparison or Wilcoxon matched-pairs signed rank test (*P < 0.05).

When DNA is damaged, the histone H2AX, is phosphorylated at S139 and recruits DNA repair proteins to sites of nascent DSBs (foci) within the nucleus [54]. To test whether AURKA inhibition induces DNA DSBs, we evaluated the effects of alisertib treatment on the formation of pH2AX^S139^ foci. Alisertib treatment induced a 3.7- and 2.5-fold increase in pH2AX^S139^ foci relative to vehicle treatment in PEO1 and in SKOV3ip2 (Figure 3A, *P* < 0.05) cells, respectively.

We next asked if inhibition of AURKA activity has a direct functional effect on error-prone NHEJ DNA repair. To answer this question, cells were treated with alisertib and then subjected to a validated functional assay for NHEJ using the *Pem1-EGFP-Ad2* plasmid digested with either *HindIII* to generate DNA DSBs with compatible overhangs or *I-SceI* to generate DSBs with incompatible ends that require nucleolytic end processing before DNA repair (Figure 3B) [55]. Inhibition of AURKA activity in PARPi-sensitive PEO1 cells with 62.5 nmol/L alisertib stimulated a 1.8- and 2.5-fold increase in end-joining of the *HindIII*- and *I-SceI*-linearized plasmid, respectively (Figure 3C, *P* < 0.05). Although the effects of alisertib were less pronounced in PARPi-resistant SKOV3ip2 cells, there was still a significant increase in end-joining of the *HindIII* and *I-SceI* DNA substrates by 1.3- and 1.4-fold, respectively (Figure 3C, *P* < 0.05).

Taken together, these data suggest that AURKA inhibition induced DNA DSBs and elevated error-prone NHEJ repair of DNA DSBs with incompatible ends in both PARPi-sensitive and -resistant ovarian carcinoma cells.

### AURKA regulates PARP activity

**Figure 4:**
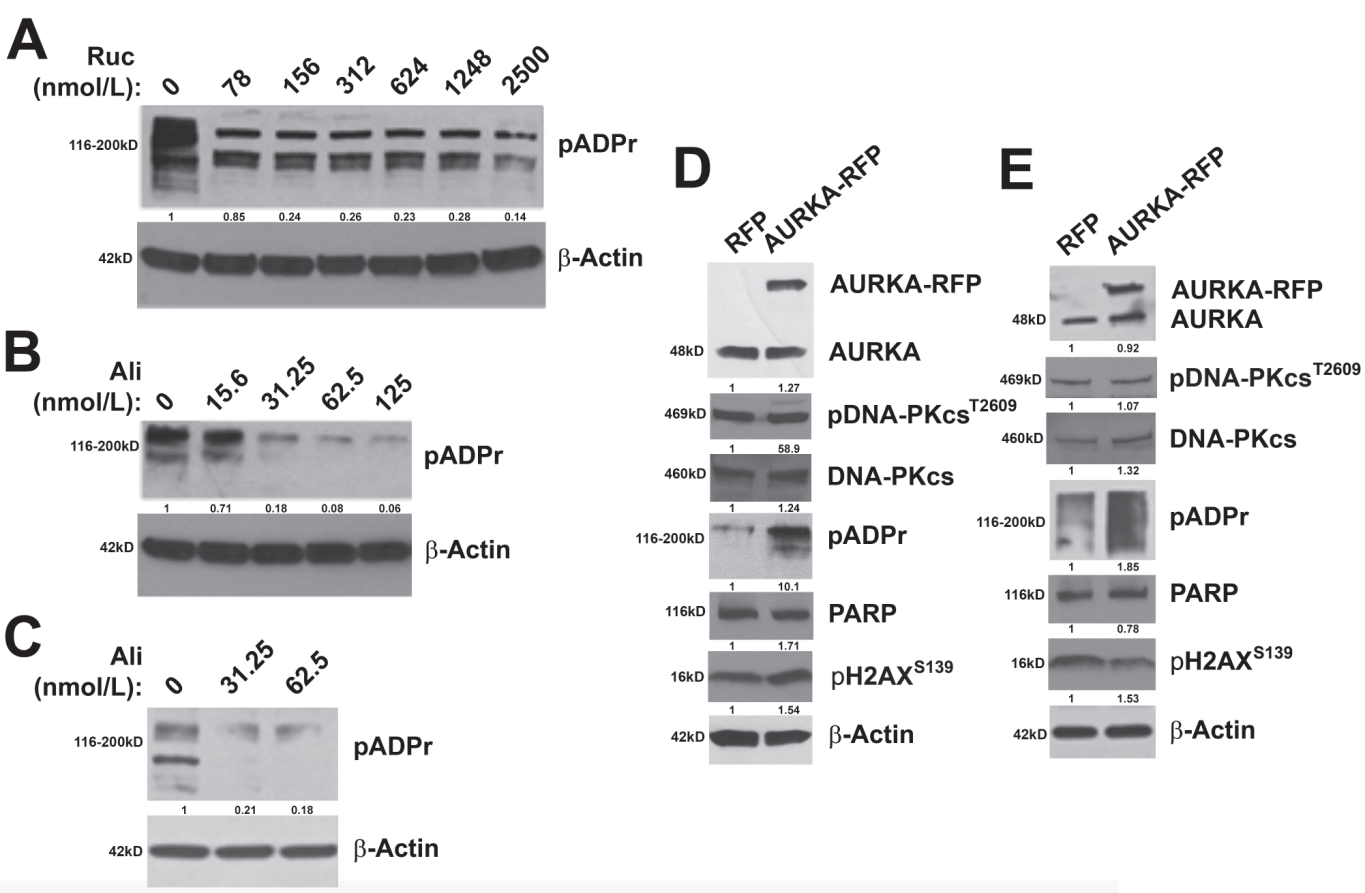
AURKA regulates PARP activity *BRCA2*- and HR-deficient **A**. PEO1 cells were treated with the PARPi, rucaparib, as a control to demonstrate that pADPr levels decrease as a result of PARP activity inhibition. **B**. PEO1 and PARPi-resistant SKOV3ip2 cells were treated with vehicle (DMSO) or increasing concentrations of alisertib (31.25, and 62.5 nmol/L) for 48 h and total protein lysates were immunoblotted with antibodies against pADPr and β-Actin (loading control). Representative immunoblots are shown. To evaluate the effects of enforced *AURKA* expression, **C**. PEO1 and SKOV3ip2 cells were transiently transfected with an *RFP* or *AURKA-RFP* expression construct for 48 h and then total protein lysates were immunoblotted with antibodies against AURKA, pDNA-PKcs^T2609^, total DNA-PKcs, pADPr, PARP, pH2AX^S139^ and β-Actin (loading control). Representative immunoblots are shown with densitometry measurements normalized to both β-actin and experimental controls under each blot.

To determine if modulation of AURKA activity alters PARP activity, we examined the effects of inhibition of AURKA activity and enforced expression of *AURKA* on the expression of pADPr polymers. Inhibition of PARP activity with PARPi decreases pADPr levels in ovarian carcinoma cells [49]. Therefore, as a control, we treated PEO1 cells with increasing concentrations of the PARPi, rucaparib, and observed the expected reduction in pADPr levels (Figure 4A). Similarly, AURKA inhibition with low nanomolar concentrations of alisertib also diminished the expression of pADPr in PEO1 and SKOV3ip2 (Figure 4B) cells, indicating diminished PARP activity. Further, enforced expression of an *AURKA-RFP* construct resulted in elevated pADPr expression relative to *RFP*-transfected cells, suggesting that PARP activity is increased in PEO1 and SKOV3ip2 (Figure 4C) cells when AURKA is overexpressed. Although pADPr levels increase, PARP levels do not change as a result of enforced expression of *AURKA* in PEO1 and SKOV3ip2 (Figure 4C) cells. In addition, pDNA-PKcs^T2609^ and pH2AX^S139^ levels were not altered as a result of AURKA overexpression (Figure 4C), suggesting that DNA DSBs are not induced and the NHEJ pathway is not stimulated.

Taken together, these observations suggest that AURKA regulates PARP activity in PARPi-sensitive and -resistant ovarian carcinoma cells.

### Alisertib treatment stimulates the NHEJ pathway and decreases PARP levels *in vivo*

**Figure 5:**
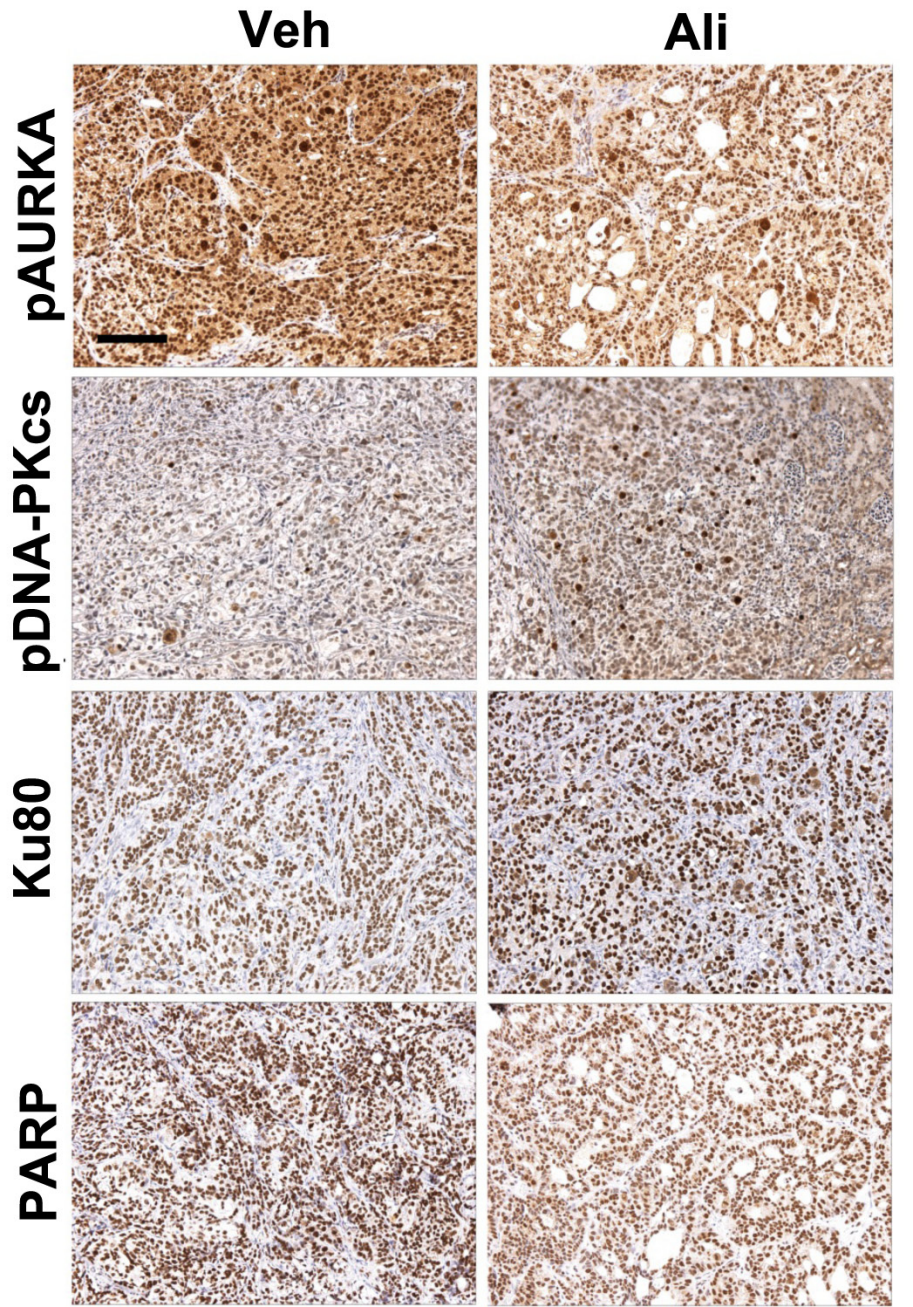
Inhibition of AURKA activity stimulates the NHEJ pathway and decreases PARP levels ***in vivo***. SKOV3ip2 cells were orthotopically implanted into SCID mice and tumor cells were allowed to grow for one month before treatment with vehicle or alisertib (10 mg/kg) to evaluate the effects of AURKA inhibition on pAURKA^T288^, pDNA-PKcs^T2609^, Ku80, and PARP levels as assayed by IHC (*n* = 5 mice/arm). Representative brightfield images captured using the same exposure times are shown.

Next, we sought to determine if AURKA regulation of the DDR and DNA repair pathways observed *in vitro* also occurs *in vivo*. To answer this question, we evaluated pAURKA^T288^, pDNA-PKcs^T2609^, Ku80, and PARP levels in SKOV3ip2 orthotopic xenografts. Briefly, SKOV3ip2 cells were implanted orthotopically by unilateral intrabursal (i.b.) injection into SCID mice (*n* = 5/arm) and tumors were allowed to develop for four weeks before treatment with vehicle or a low dose of alisertib, 10 mg/kg (four cycles of 5 days on/2 days off), to ensure that observations are not due to off-target effects. Mice were euthanized six hours after the final administration of alisertib and expression of PARP and signaling components in the NHEJ pathway were evaluated by immunohistochemistry (IHC). Detection of pAURKA^T288^ (H-score = 300 vs 200) showed that AURKA activity is decreased in alisertib-treated tumors relative to vehicle-treated tumors confirming drug activity (Figure 5). Notably, we observed elevated pDNA-PKcs^T2609^ levels (H-score = 100 vs 200, strong positive staining nuclei = 17 vs 43) and a modest increase in Ku80 expression (H-score = 200 vs 300) in alisertib-treated tumors compared to controls (Figure 5), suggesting stimulation of the NHEJ pathway. Conversely, we detected a decrease in PARP (H-score = 300 vs 200) levels in alisertib-treated tumors relative to vehicle-treated control tumors (Figure 5). Collectively, these observations suggest that AURKA inhibition decreases PARP expression and stimulates the NHEJ DNA repair pathway in PARPi-resistant ovarian tumor cells *in vivo*, consistent with our *in vitro* observations.

### Alisertib stimulates BRCAness and synergy to PARP inhibition

**Figure 6:**
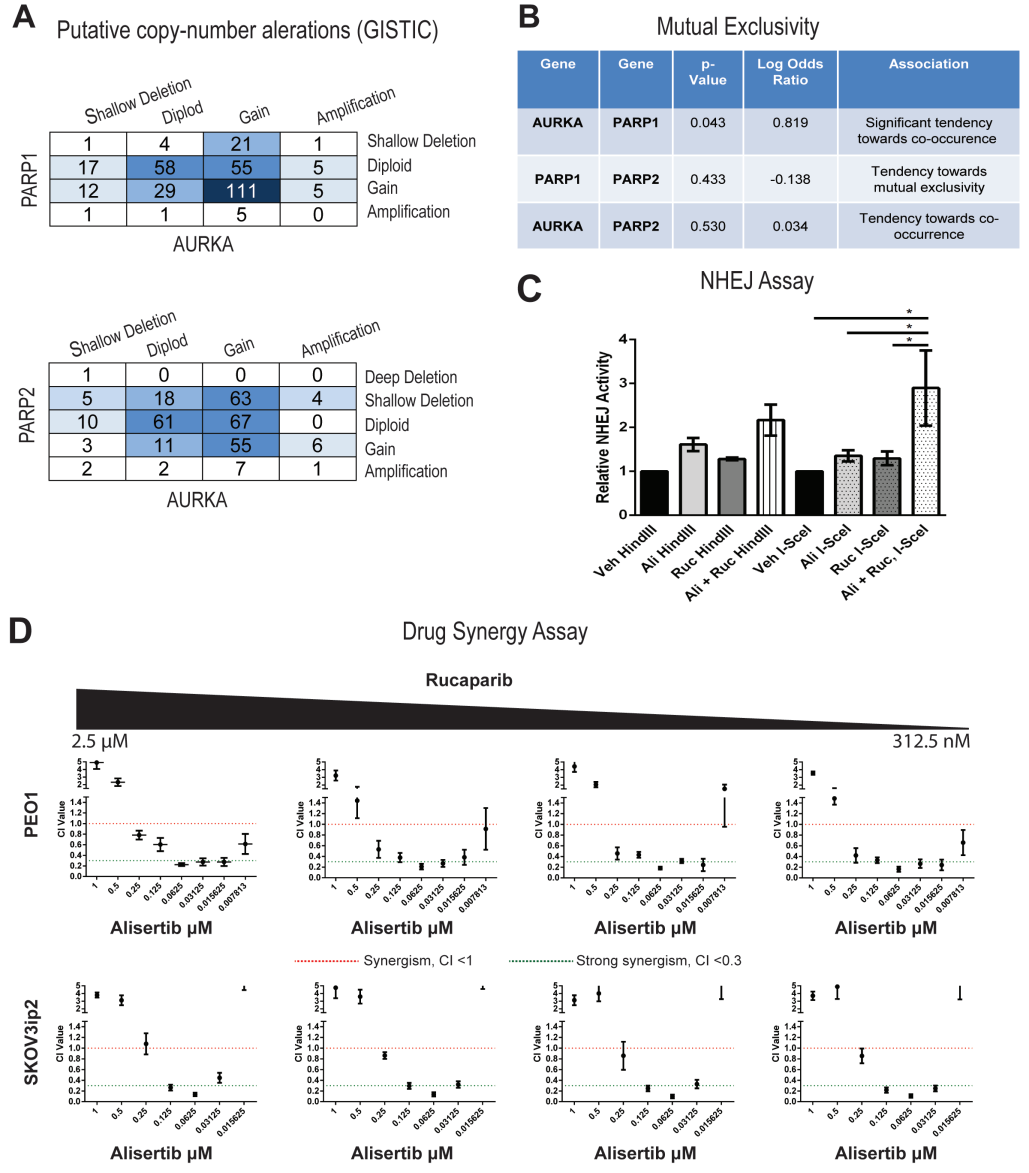
Drug synergy between alisertib and rucaparib in both PARPi sensitive and resistant cell lines **A**. Copy number alterations are similar between *AURKA* and *PARP1* as well as between *AURKA* and *PARP2*. **B**. Mutual exclusivity analysis suggest a significant association between *AURKA* and *PARP1* and a trend of co-occurrence between *AURKA* and *PARP2*. **C**. Combinations of alisertib (31.25 nM) and rucaparib (313 nM) significantly enhance the NHEJ function in PEO1 cell lines compared to vehicle, alisertib alone (31.25 nM), or rucaparib alone (313 nM) (*n* = 5, *= *P<0.05*, two-way ANOVA). **D**. Combination index values for both PEO1 and SKOV3ip2 cells between rucaparib (2.5 μM, 1.25 μM, 625 nM, and 312 nM) and eight two-fold serial dilutions of alistertib (1 μM to 7.8 nM). CI values were considered either synergistic (CI< 1.0, red line) or strongly synergistic (CI< 0.3, green line). Each data point is represented of three independent experiments and plotted as mean ± SD.

While PARP inhibitors have been considered a breakthrough treatment in ovarian cancer, they are generally only recommended in *BRCA* mutant tumors. Finding clinical targets that can enhance “BRCAness”, like we have shown with AURKA inhibition, could open up a whole new cohort of patients that might benefit from PARP inhibitors. In a query of over 400 ovarian cancer tumors in TCGA [56], using the cBioPortal (www.cbioportal.org), we determined that copy number alterations of *PARP1* and *AURKA* were often observed in the same tumors (Figure 6A) and observed a significant tendency towards co-occurrence in a mutual exclusivity analysis (Figure 6B, *P= 0.043*). In contrast, co-mRNA expression of both genes was not significantly correlated (data not shown). These genomic data suggest exploring a combination of alisertib and rucaprarib might have clinical benefit.

Synthetic lethality is the simultaneous alteration of two genes or proteins which causes cell death, while alteration of either gene/protein alone does not. Since we observed that AURKA inhibition leads to BRCAness by decreasing BRCA1/2 protein levels, we hypothesized that the combination of alisertib and rucaparib would be synergistic. First, using the functional assay for NHEJ shown in Figure 3, PEO1 cells treated with the combination of alisertib (31.25 nM) and rucaparib (313 nM) lead to a 1.3-fold increase in end-joining of the *I-SceI*-linearized plasmid relative to alisertib or rucaparib alone and a 2.9-fold increase versus vehicle (Figure 6C, *P* < 0.05). The enhancement in repair was also observed for the drug combination when using the *HindIII* assay, but the change was not deemed statistically significant. Next we evaluated the *in vitro* activities of the drug combination in both the *BRCA* wild-type (SKOV3IP) and *BRCA* defective (PEO1) cells using a combination matrix of rucaparib (2.5 μM, 1.25 μM, 625 nM, and 312 nM) and eight two-fold serial dilutions of alistertib (1 μM to 7.8 nM), with triplicate sampling. As shown in Figure 6D, the combined treatment of alisertib and rucaparib inhibited cell growth and was synergistic (CI < 1.0, red line) or strongly synergistic (CI < 0.3, green line) across both cell lines.

Taken together, these results suggest that induction of a BRCAness phenotype by alisertib, as a result of decreased BRCA protein expression and induction of NHEJ DNA repair, could help sensitize patient tumors without HR defects (*e.g*., wild-type for *BRCA1/2*) to PARPi. Importantly, this clinical approach could improve the efficacy and decrease the adverse side effects of both alisertib and PARPi, since the activity of the combination was more pronounced at lower dosages.

## DISCUSSION

Although it is known that AURKA regulation of mitosis and centrosome function plays an important role in OC biology [7–13], there remains a large gap in our knowledge of alternative, non-mitotic functions of this kinase in tumor cells. In the current study, we investigated the relatively undefined role of AURKA in mediating the DDR and DNA repair in ovarian carcinoma cells and discovered that AURKA regulates the activity of PARP and DNA-PKcs, a key signaling molecule in the error-prone NHEJ pathway.

**Figure 7:**
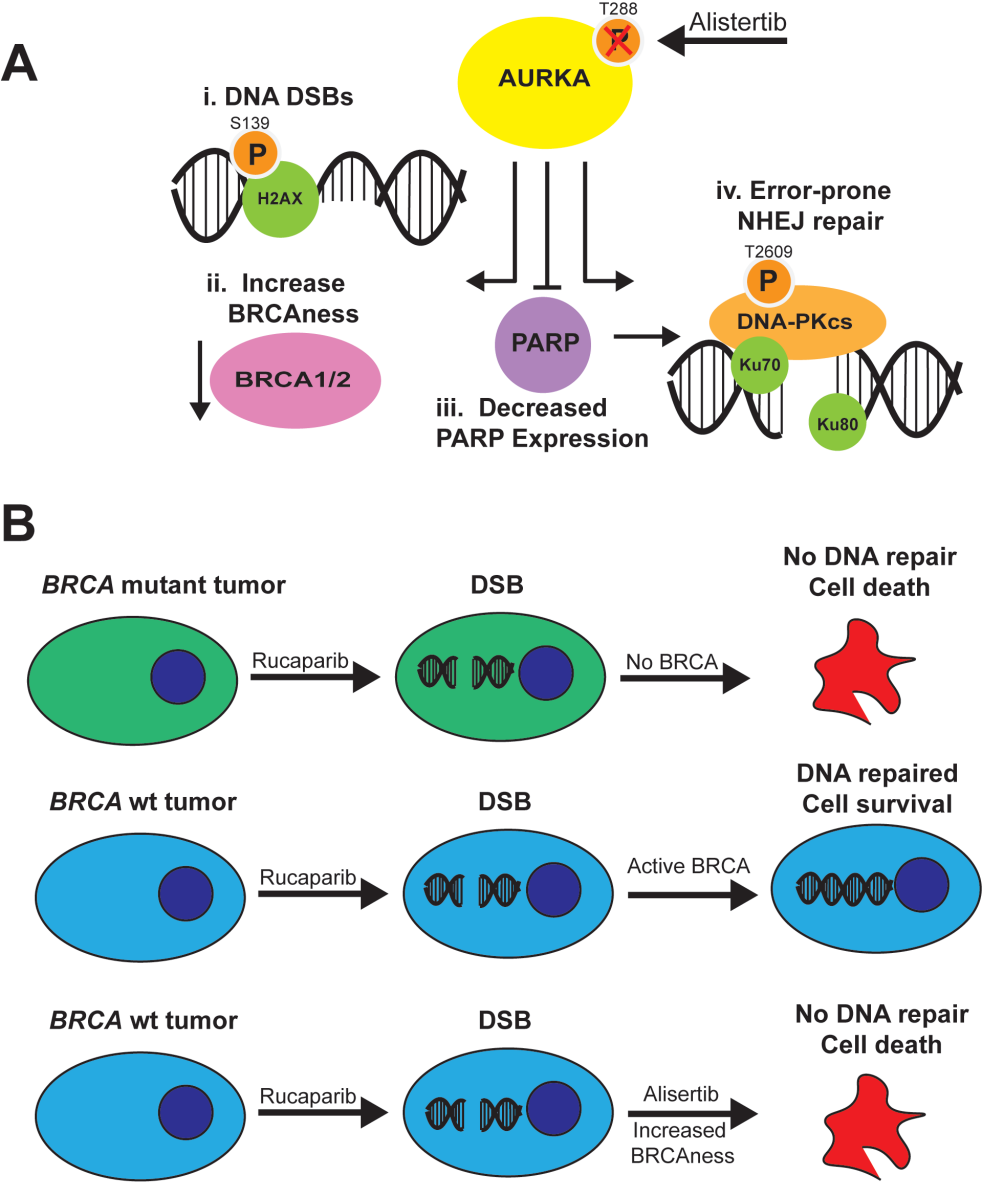
Model for effects of AURKA inhibition on NHEJ in ovarian carcinoma cells and combination with PARP inhibitors **A**. Inhibition of AURKA activity stimulates error-prone NHEJ DNA repair of DNA DSBs with incompatible ends, which may result in genomic instability that is lethal to tumor cells. AURKA inhibition in ovarian carcinoma cells i) induces DNA DSBs as evidenced by an increase in gH2AX foci, ii) increases BRCAness as a result of decreased BRCA1 or BRCA2 levels, iii) decreases PARP expression and activity, which would relieve PARP suppression of Ku function, and iv) stimulates DNA-PKcs activity. The increase in BRCAness, decrease in PARP activity and stimulation of DNA-PKcs activity would favor the use of the more error-prone NHEJ pathway and combination therapy with PARP inhibitors. **B**. Rucaparib, a PARP inhibitor, is effective in *BRCA*^mut^ tumors (top) but not *BRCA*^wt^ tumors (middle) which repair DNA double strand breaks. Combination of rucaparib with alisertib, which induces a BRCAness phenotype, increases the function of PARPi in BRCAwt tumors (bottom).

Based on our findings, we propose a model in which inhibition of AURKA activity promotes NHEJ DNA repair through distinct mechanisms (Figure 7A). First, AURKA inhibition induces the formation of pH2AX^S139^ foci (i) in the nucleus, similar to the effects of PARPi on ovarian carcinoma cells [49]. AURKA inhibition also increases BRCAness by decreasing BRCA1/2 protein levels (ii) and stimulates the error-prone NHEJ pathway by diminishing PARP expression (iii) and increasing DNA-PKcs activity (iv). Since PARP and Ku directly compete for the binding of DNA ends [57], a decrease in PARP levels should favor Ku binding to DNA ends. All of these changes are predicted to favor the NHEJ repair pathway over the HR pathway [57].

Treatment with the PARPi, veliparib, resulted in distinct effects on HR-deficient versus HR-proficient ovarian carcinoma cells. Veliparib treatment increased pH2AX^S139^ and pDNA-PKcs^T2609^ levels in HR-deficient (PARPi-sensitive) but not HR-proficient (PARPi-resistant) ovarian carcinoma cells [49]. Also, PARPi stimulation of NHEJ repair resulted in genomic instability which was cytotoxic to HR-deficient but not -proficient cells. The same study demonstrated that RNAi-mediated silencing of *PARP* expression significantly reduced colony formation in HR-deficient cells, but had no effect on colony formation in HR-proficient cells [49].

In contrast to these findings for PARPi, we discovered that inhibition of AURKA activity with alisertib significantly diminished the growth and clonogenic survival of PARPi-sensitive and -resistant ovarian carcinoma cells. Alisertib treatment also decreased PARP expression and activity and activated DNA-PKcs, thereby stimulating error-prone NHEJ repair of DSBs with incompatible ends in both PARPi-sensitive and -resistant cells. To our knowledge, this study is the first to demonstrate that inhibition of AURKA activity increases error-prone NHEJ repair. These findings suggest that alisertib may be used in combination with PARPis to sensitize PARPi-resistant or otherwise weakly sensitive ovarian carcinoma cells to PARP inhibition, although we cannot rule out the possibility that alisertib treatment decreases ovarian carcinoma cell viability because other AURKA-regulated functions not related to DNA repair, are altered. Inhibition of AURKA activity also decreased the expression of BRCA1/2 in ovarian carcinoma cells. BRCA1 expression changes throughout the cell cycle, with highest expression observed during S phase, and high levels are maintained until G2/M before decreasing in early G1 [58, 59]. BRCA2 expression is lowest during G_0_ and early G_1_ and increases as cells enter S phase [60–63]; there are reports of roles for BRCA2 during G2/M [64, 66] and mitosis [66–71]. We do not know why BRCA1/2 expression decreased as a result of alisertib treatment, but this phenomenon might be able to be exploited to provide options for women whose tumors are wild-type for *BRCA1/2* and related DNA repair genes and are not strong candidates for treatment with PARP inhibitors. One possible explanation is that AURKA activity may be required for the stability of BRCA1/2, since a number of studies report a physical and/or functional relationship between AURKA and BRCA1/2 [27, 28, 72, 73].

The modest effects of alisertib in clinical trials with platinum-resistant and -refractory patients may reflect the gap in our knowledge of the non-mitotic functions of AURKA. Elucidation of AURKA-regulated pathways that mediate the DDR and DNA repair may identify biomarkers for predicting response to molecular therapeutics which target these pathways and may also uncover novel targets for combination therapy strategies utilizing PARPi. In this paper, we showed that exploring the combination of AURKAi and PARPi showed singificant drug synergy and could open up the use of PARP inhibitors in non-*BRCA* mutant tumors (Figure 7B).

## MATERIALS AND METHODS

### Cell culture and transfection

The human ovarian carcinoma cell lines and culture medium used were: PEO1 A2780, MDAH, OVCAR5, OVCAR10 and PEO4 (kind gift of Dr. Michael White, University of Texas Southwestern) in RPMI supplemented with 10% FBS and insulin (0.25 units/mL); SKOV3ip2 [17] in McCoy's 5A supplemented with 10% FBS, non-essential amino acids and 800 mg/mL G418; OVCA429 in MEM supplemented with 10% FBS, non-essential amino acids and sodium pyruvate. All media was supplemented with penicillin/streptomycin (100 mg/mL). The plasmid constructs, pcDNA3.1-*AURKA-RFP* and pcDNA3.1-*RFP* (generous gift of Dr. Erica Golemis, Fox Chase Cancer Center), were transiently transfected (4 μg DNA) using Lipofectamine 2000 (Thermo Scientific, Rockford, IL) or TransIT-LT1 (Mirus Bio, Madison, WI) according to the manufacturer's instructions. The effect of RNAi-mediated *AURKA* knockdown was assayed in cells transfected twice (24 h apart) with 25 nmol/L (for SKOV3ip2) or 50 nmol/L (for PEO1) of *AURKA*-specific siRNA or the AllStars Negative Control siRNA (Qiagen, Valencia, CA; oligo sequence is proprietary) using Lipofectamine 2000. The sequences for *AURKA*-specific siRNA oligos utilized are: 5’-AAUAUUAGGAUGCCGAAGGTG-3’ (si1); 5’-UAAAUGGAGGUAGAGCACGTG-3’ (si2).

### Drug treatment, cell viability and clonogenic assays

Cells were plated in 96-well black, clear bottom plates (Greiner Bio-One, Monroe, NC) and treated the following day with alisertib (Selleck Chemicals, Houston, TX). For each experiment, cells were treated in quadruplicate with vehicle (DMSO) or alisertib (0.00781, 0.0156, 0.0313, 0.0625, 0.125, 0.250, 0.50 and 1.0 μmol/L) for 7 days, and then viability was evaluated using the Cell Titer Blue assay (Promega, Madison, WI) by measuring fluorescent intensity on the Tecan M200 plate reader (Tecan US, Inc., Morrisville, NC) according to the manufacturer's instructions. For PARPi treatment, cells were treated in quadruplicate with increasing concentrations of rucaparib (0.0781, 0.156, 0.313, 0.625, 1.25, 2.5, 5.0, and 10.0 mmol/L) [Selleck Chemicals] for 7 d. Nonlinear regression analysis was performed using GraphPad Prism version 5.01 (GraphPad Software, La Jolla, CA) to generate a log (inhibitor) versus drug response curve with variable slope and to calculate the IC_50_ ± SEM.

Clonogenic survival assays were performed by plating PEO1 (1000 cells/dish) and SKOV3ip2 (200 cells/dish) cells on 60 mm dishes and treating the next day with either vehicle (DMSO) or alisertib (0.0313, 0.0625, 0.125, and 0.250 μmol/L) for three days. Cells were then rinsed with fresh media and SKOV3ip2 and PEO1 cells were cultured in complete media for nine and ten days, respectively. Colonies (^3^ 50 cells) were fixed with 4% NBF for 10 min, stained with crystal violet for 30 min, and rinsed excessively with water. The plate was divided into four fields and the mean number of colonies per field ± SEM was calculated.

### Immunoblotting

Cell monolayers were lysed in Mammalian Protein Extraction Reagent (MPER™, Thermo Scientific) supplemented with Halt™ Phosphatase Inhibitor Cocktail (Thermo Scientific) and Complete™ Mini Protease Inhibitor Cocktail (Roche Diagnostics, Indiannapolis, IN). Protein concentrations of cell lysates were determined using the BCA Protein Assay (Thermo Scientific) and proteins were resolved using 7.5%, 12%, or 4-20% gradient SDS-PAGE gels (Bio-Rad, Hercules, CA). Proteins were transferred to supported nitrocellulose membrane overnight at 25 V, and membranes were blocked in 5% non-fat dry milk or 5% BSA in TBST, incubated overnight at 4 C with primary antibody in block, followed by Immun-Star HRP-conjugated secondary antibody (Bio-Rad) at 1:10,000; signal was detected with SuperSignal West Pico Chemiluminescent Substrate (Thermo Scientific). Antibodies directed against the following were used: p-DNA-PKcs^T2609^ (Thermo Scientific), DNA-PKcs (Santa Cruz Biotechnology, Dallas, TX); AURKA, BRCA1, PARP (Cell Signaling, Danvers, MA); BRCA2 (R & D Systems, Minneapolis, MN), pADPr or PAR (BD Biosciences, San Jose, CA), pH2AX^S139^ (Calbiochem), and β-Actin (Sigma-Aldrich). Pixel densities of blot images were calculated using Image-J software (NIH). Changes in protein levels were normalized to loading controls and expressed as fold change relative to treatment controls.

### Immunofluorescent staining and microscopy

Cells were cultured on chamber slides and then treated with alisertib (62.5 nmol/L) for 24 h before incubating with extraction buffer (20 mmol/L HEPES, pH 7.5, 20 mmol/L NaCl, 5 mmol/L MgCl_2_, 0.5% Nonidet P-40), on ice for 20 min, then fixed in 4% NBF for 15 min, and permeabilized in 0.5% Triton-X100/PBS for 10 min at room temperature. Cells were blocked in 5% normal goat serum in TBS for 1 h at room temperature and then incubated with anti- pH2AX^S139^ diluted in block overnight at 4°C and then with goat-anti-mouse Dylight 488-conjugated secondary antibody for 1 h at room temperature. Immunofluorescent images were acquired using a Nikon Eclipse 80i microscope (Melville, NY) and digital camera with Metamorph software v. 7.7 (Molecular Devices, Sunnyvale, California) using identical exposure times. The number of nuclei with ≥ 10 pH2AX^S139^-positive foci was enumerated in five different fields for each treatment condition and the mean percentage of nuclei with ≥ 10 pH2AX^S139^ foci ± SE was calculated. Statistical analysis was performed with GraphPad Prism using Wilcoxon matched-pairs signed rank test; *P* < 0.05 was considered significant.

### Nonhomologous end-joining DNA repair assay

Cells were treated with 5 μmol/L QVD-OPhe (Sigma-Aldrich), a broad spectrum caspase inhibitor, before being transfected twice (24 h apart) with 2 μg of *pem1-Ad2-EGFP* plasmid (generous gift of Dr. Eric A. Hendrickson, University of Minnesota Medical School) digested with either *HindIII* (Thermo Scientific) or *I-SceI* (New England Biolabs, Ipswich, MA), and 0.5 μg of *pcDNA3.1-RFP* plasmid using Lipofectamine 2000. Cells were then treated with either vehicle (DMSO) or 62.5 nmol/L alisertib for 48 h before analysis on the Becton Dickinson LSRII flow cytometer (Franklin Lakes, NJ) to detect GFP^+^ cells, which have successfully repaired DNA DSBs. The number of GFP^+^ cells was normalized to the number of RFP^+^ cells as a control for transfection efficiency, and statistical analysis was performed with GraphPad Prism using either one-way ANOVA followed by Tukey's multiple comparison or by Wilcoxon matched-pairs signed rank test; *P* < 0.05 was considered significant.

### RNA isolation and real-time PCR analyses

PEO1 cells were cultured in a 60 mm dish and treated with 62.5 nmol/L alisertib for 16 or 48 h before RNA isolation using the RNeasy Mini Kit (Qiagen). Genomic DNA was removed before cDNA was synthesized from 1 mg RNA using the Quantifect Reverse Transcription Kit (Qiagen), according to the manufacturer's instructions. Real-time PCR was performed in quadruplicates to assay the expression of *BRCA1*, *PARP1*, and *53BP1* mRNA using Applied Biosystems^TM^ Taqman^®^ Assays (ThermoFisher Scientific), according to the manufacturer's instructions, and the Bio-Rad CFX96^TM^ real-time detection system and accompanying software to analyze normalized relative mRNA expression and to perform statistical analyses. *RPS13* was used as a control for normalizing gene expression *P* < 0.05 was considered significant.

### Orthotopic xenografts and immunohistochemistry

The University of Kansas Medical Center Institutional Animal Care and Use Committee (IACUC) approved all procedures involving mice. Seven to fourteen week-old female C.B-17 severe combined immunodeficient (SCID) mice (Harlan Research Models, Indiannapolis, IN) were used for intrabursal (i.b.) injections as described [74]. Mice were given unilateral (left side) i.b. injections of SKOV3ip2-Luc-D3 cells (1 × 10^6^), also referred to as SKOV3ip2 [17]. Alisertib (Selleck Chemicals) was suspended in 10% 2-hydroxypropyl-b-cyclodextrin (Sigma-Aldrich) with 1% sodium bicarbonate and 10 mg/kg administered orally to mice using a 5 day on/2 day off schedule. Mice were euthanized by CO_2_ inhalation, necropsied and examined for gross abnormalities. Paraffin-embedded tissue sections were used for immunohistochemical (IHC) staining with antibodies directed against pAURKA^T288^ (Bethyl Laboratories, Montgomery, TX); pDNA-PKcs^T2609^ (Thermo Scientific); and Ku80 and PARP (Cell Signaling). Brightfield images of IHC staining were acquired on a Nikon Eclipse 80i microscope and digital camera using identical exposure times. The level of expression of each antigen was calculated using a semi-quantitative approach for IHC staining, *i.e.,* H-score: a summation of the percentage of area stained (0 to 100) at each intensity level multiplied by the weighted intensity (*e.g*., 1, 2, or 3; where 0 is no staining, 1 is weak staining, 2 is moderate staining and 3 is strong staining) [75]. For pDNA-PKcs^T2609^ the average number of positively staining nuclei per high powered field were compared in alisertib and vehicle treated tumors.

### Drug combination assays

Drug combination studies were performed using the combination index (CI) method described by Chou and Talalay [76]. Cells from PEO1 or SKOV3ips cell lines were plated overnight and treated with rucaparib (2.5 μM, 1.25 μM, 625 nM, and 312 nM) and eight two-fold serial dilutions of alisertib (1 μM to 7.8 nM) in a combination matrix. Assays were performed as biological triplicate using triplicate wells within each experiment. Cell viability following 48 h of treatment from the serial dilutions was evaluated using CellTiter-Blue as previously described and the viability data were then analyzed using CalcuSyn (version 2.1, BioSoft, UK) to calculate the synergy between the two drugs at each molar ratio evaluated. Drug combinations which yielded CI values less than 1 were considered to be synergistic as previously reported [77, 78] and used in our laboratory [79, 80].

## SUPPLEMENTARY MATERIALS FIGURES AND TABLE


