# Supplementary material for: Aurora A kinase regulates non-homologous end-joining and poly(ADP-ribose) polymerase function in ovarian carcinoma cells
Source: Oncotarget. 2017 Jul 5;8(31):50376–92. doi: 10.18632/oncotarget.18970 (PMC5584138; doi:10.18632/oncotarget.18970)
Supplement: Supplementary file 1 [file oncotarget-08-50376-s001.pdf]

Aurora A kinase regulates non-homologous end-joining and poly(ADP-ribose) polymerase function in ovarian carcinoma cells

Supplementary Materials

Cell culture

The human ovarian carcinoma cell lines and culture medium used were: PEO1, PEO4, A2780, MDAH, OVCAR5, and OVCAR10 in RPMI supplemented with 10% FBS and insulin (0.25 units/mL); SKOV3ip2[15] in McCoy's 5A supplemented with 10% FBS, non-essential amino acids and 500 µg/mL G418; OVCA429 in MEM supplemented with 10% FBS, non-essential amino acids and sodium pyruvate. All media was supplemented with penicillin/streptomycin (100 µg/mL).

Rucaparib treatment and viability assay

For each experiment, cells were cultured in 96-well black, clear bottom plates (Greiner Bio-One) and treated in quadruplicate with increasing concentrations of rucaparib (0, 0.0781, 0.156, 0.313, 0.625, 1.25, 2.5, 5.0, and 10.0 µmol/L) for 7 d before assaying cell viability with Cell Titer Blue (Promega) by measuring fluorescent intensity on the Tecan M200 plate reader (Tecan US, Inc.) according to the manufacturer's instructions. Cells were also treated with vehicle (DMSO) as a control. Nonlinear regression analysis was performed using GraphPad Prism (GraphPad Software) to generate a log(inhibitor) versus drug response curve with variable slope and to calculate the $IC_{50} \pm SEM$.

RNA isolation and real-time PCR analyses

PEO1 cells were cultured in a 60 mm dish and treated with 62.5 nmol/L alisertib for 16 or 48 h before RNA isolation using the RNeasy Mini Kit (Qiagen). Genomic DNA was removed before cDNA was synthesized from 1 µg RNA using the Quantifect Reverse Transcription Kit (Qiagen), according to the manufacturer's instructions. Real-time PCR analyses were performed to assay the expression of *BRCA1, PARP1*, and *53BP1* mRNA using Applied Biosystems™ Taqman® Assays consisting of primers for the target genes and primer-specific probes (ThermoFisher Scientific) and the Bio-Rad CFX96™ real-time detection system and accompanying software to evaluate normalized relative mRNA expression and statistical analyses. *RPS13* was used as a control for normalizing gene expression.

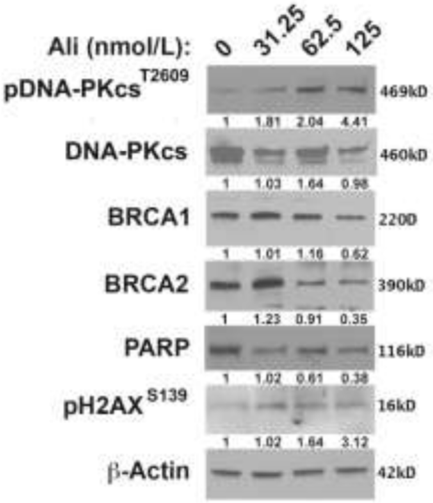

Supplementary Figure 1: Inhibition of AURKA activity stimulates the NHEJ pathway and decreases the expression of proteins required for HR. To determine the effects of inhibiting AURKA activity on NHEJ or HR-mediated DNA repair pathways in ovarian carcinoma cells exhibiting intermediate sensitivity to the PARPi, rucaparib, OVCA429 cells were treated with alisertib (0, 31.25, 62.5, or 125 nmol/L) and then total protein lysates were immunoblotted with antibodies against pDNA-PKcs^{T2609}, total DNA-PKcs, BRCA1, BRCA2, PARP1, pH2AX^{S139}, and β-Actin (loading control). Cells were treated with vehicle (DMSO) as a control. Representative immunblots are shown with densitometry measurements normalized to both β-actin and experimental controls under each blot.

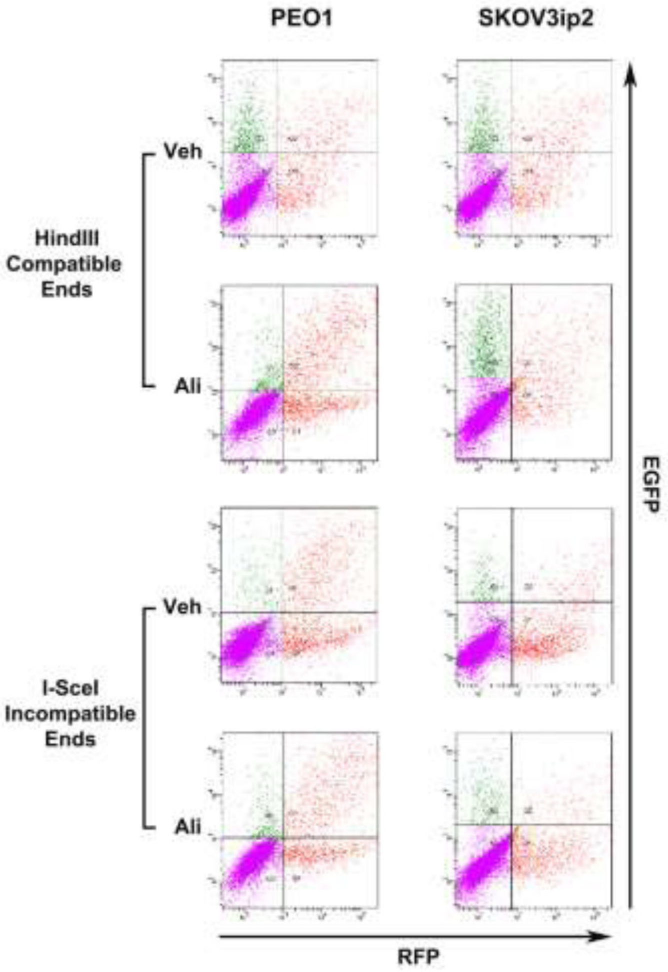

Supplementary Figure 2: Inhibition of AURKA activity stimulates NHEJ in ovarian carcinoma cells. Representative dot plots of (A) PEO1 and (B) SKOV3ip2 cells assayed for NHEJ by flow cytometry. Cells were transfected with the NHEJ assay plasmid, *Pem1-Ad2-EGFP* digested with either *HindIII* or *I-SceI* to generate DNA DSBs with compatible overhangs or incompatible ends, respectively, and then treated with either vehicle (DMSO) or 62.5 nmol/L alisertib for 48 h. An *RFP* plasmid was co-transfected with *Pem1-Ad2-EGFP* to as a control for transfection efficiency among different treatment conditions. The total number of $EGFP^+$ cells was normalized to the total number of RFP^+ cells to evaluate end-joining for the *HindIII* and *I-SceI* DNA substrates.

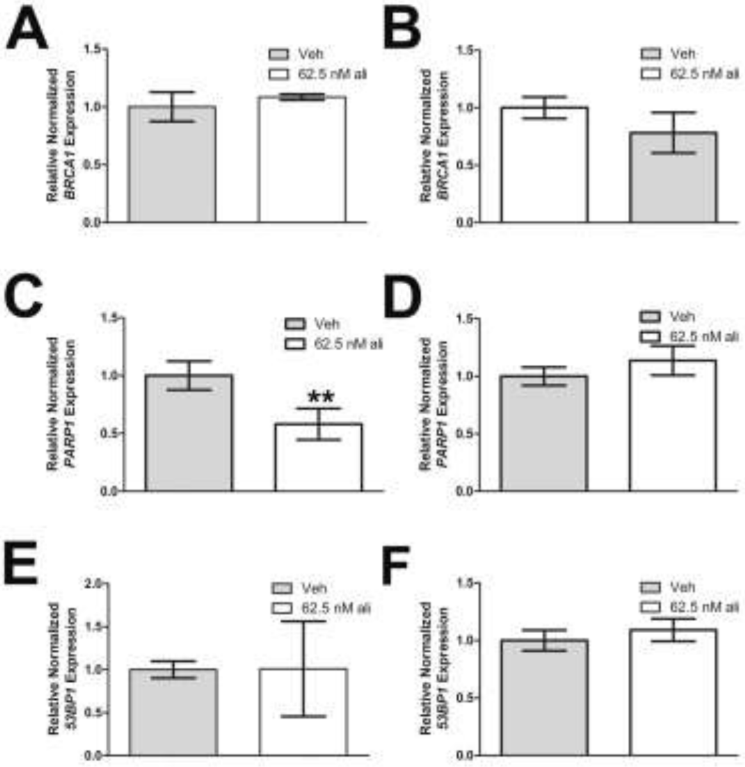

Supplementary Figure 3: Inhibition of AURKA activity decreases *PARP1*, but not *BRCA1/2 mRNA* levels. The *PARPi*-sensitive ovarian carcinoma cells, PEO1, were treated with either vehicle (DMSO) or 62.5 nmol/L alisertib for 16 or 48 h and then RNA was isolated for qPCR analysis of *PARP1*, *BRCA1*, and *BRCA2* mRNA expression using TaqMan primer probes specific for each gene and an internal control gene, *RPS13*. Graphs depicting mean normalized relative gene expression ± SEM are shown (*, $P < 0.05$).

Supplementary Table 1: IC_{50} values for rucaparib

Cell Line	Rucaparib IC_{50} (μmol/L ± SE)
PEO1	0.324 ± 0.136
MDAH	2.00 ± 0.880
A2780	2.05 ± 0.620
OVCAR5	5.13 ± 1.26
OVCAR10	5.20 ± 1.64
OVCA429	5.50 ± 1.42
PEO4	NA[a]
SKOV3ip2	NA[a]
HOSE	NA[a]

[a]Cells are resistant to rucaparib and an IC_{50} value cannot be calculated.
